# Supplementary figures and images for: PPDPF is not a key regulator of human pancreas development
Source: PLoS Genet. 2025 Apr 7;21(4):e1011657. doi: 10.1371/journal.pgen.1011657 (PMC12037078; doi:10.1371/journal.pgen.1011657)

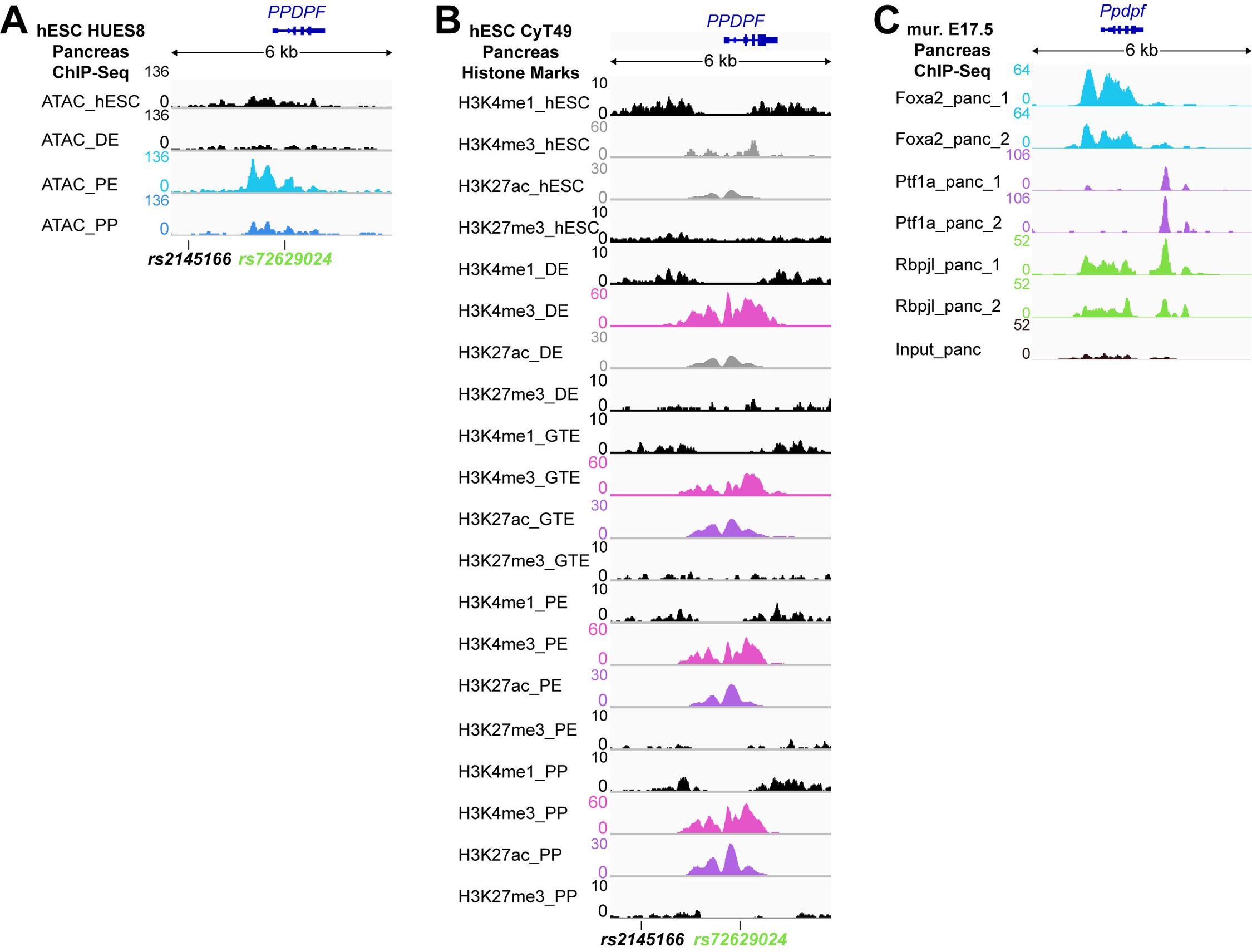

Supplement: S1 Fig — (A) ATAC-seq (Assay for Transposase-Accessible Chromatin using sequencing)-based chromatin opening at PPDPF locus from our previous in vitro differentiation of the hESC line HUES8 [13]. (B) Histone marks along the time course of differentiation in CyT49 differentiation complementing main Fig 2F. (C) Chromatin immunoprecipitation (ChIP)-seq binding peaks of murine Foxa2, Ptf1a, and Rbpjl from publicly available ChIP-seq data at E17.5 [75]. ATAC- and ChIP-seq peaks have been visualized via Integrative Genomics Viewer (IGV) [54]. (TIF) [file pgen.1011657.s001.tif]

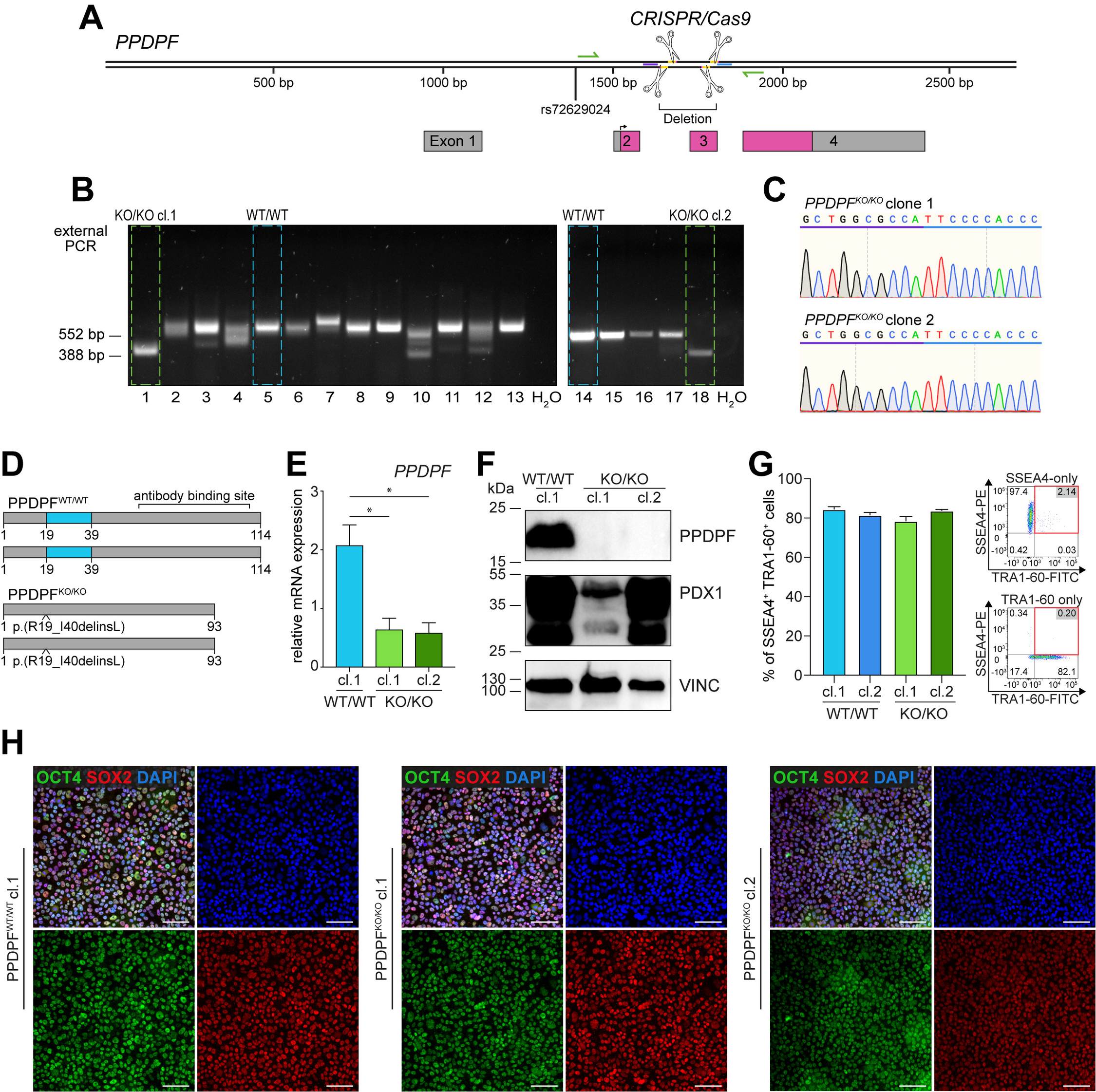

Supplement: S2 Fig — (A) Scheme of the applied four times CRISPR/Cas9 nicking strategy to delete exon 3 of PPDPF. (B) PCR-based clone screening after gene editing in the hESC line HUES8 highlighting the two employed PPDPFKO/KO clones. Note that the PCR band size, which was generated with primers spanning the target region, decreases after successful gene editing (from around 550 bp to around 400 bp). (C) Sanger sequencing of the two PPDPFKO/KO clones. Both clones harbored identical homozygous mutations. (D) Predicted alterations on protein level caused by the induced genetic deletion. (E) PPDPF mRNA expression relative to HMBS in PPDPFKO/KO and PPDPFWT/WT PPs (n = 3; Mean ± SEM; ordinary one-way ANOVA followed by Dunett’s multiple comparison test; cl.: clone). (F) Western Blot at PE stage confirming KO of PPDPF on protein level (200 µg, n = 2). (G) Flow cytometry analysis of pluripotency marker SSEA4 and TRA1-60 in PPDPFKO/KO and PPDPFWT/WT hESCs (n = 1, in duplicates; Mean ± SD). (H) ICC/IF staining of pluripotency marker OCT4 and SOX2 in PPDPFKO/KO and PPDPFWT/WT hPSCs (scale bar: 100 µm, n = 1). (TIF) [file pgen.1011657.s002.tif]

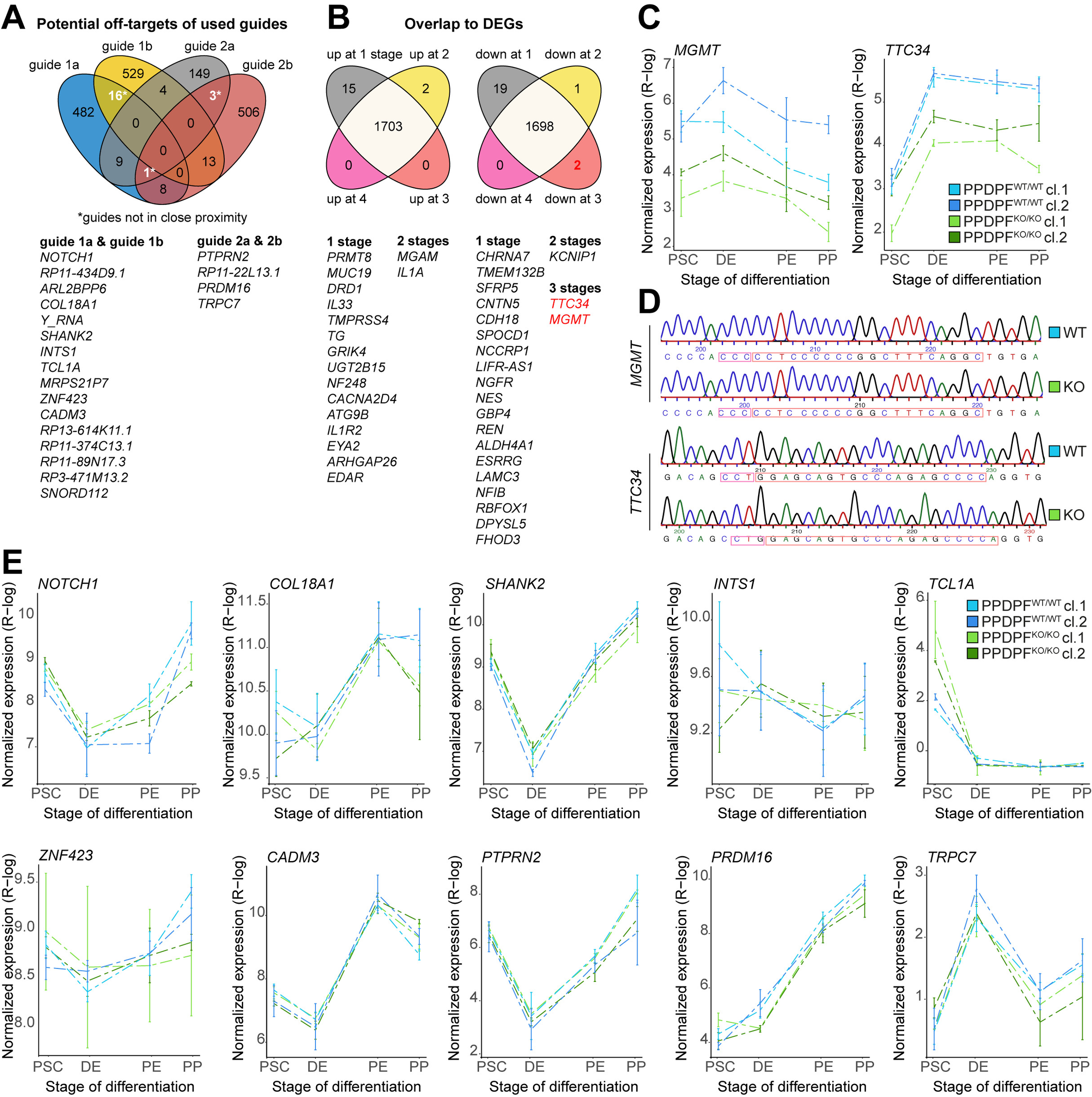

Supplement: S3 Fig — (A) Overlap between CRISPOR [24] predicted potential off-targets of different guides. Two nicks on complementary strands and in close proximity are required for induction of a double strand break (DSB). (B) Overlap between predicted potential off-targets and RNA-seq DEGs. Only 2 genes were found, of which expression was significantly reduced in at least 3 stages. (C) Line plots depicting Mean ± SEM of the two genes, MGMT and TTC34, with (D) subsequent sanger sequencing on the potential off-target sites. Sequences were unaltered in all clones. One representative PPDPFWT/WT and PPDPFKO/KO hESC clone sequence is shown. (E) Exemplary line plots of genes (Mean ± SEM), in which potential off-targets were shared between guide1a and 1b, respectively 2a and 2b. Also, other pairwise comparisons (e.g. guide 1a with 2a) were investigated with no indications for a loss- or gain-of-transcript mutation. (TIF) [file pgen.1011657.s003.tif]

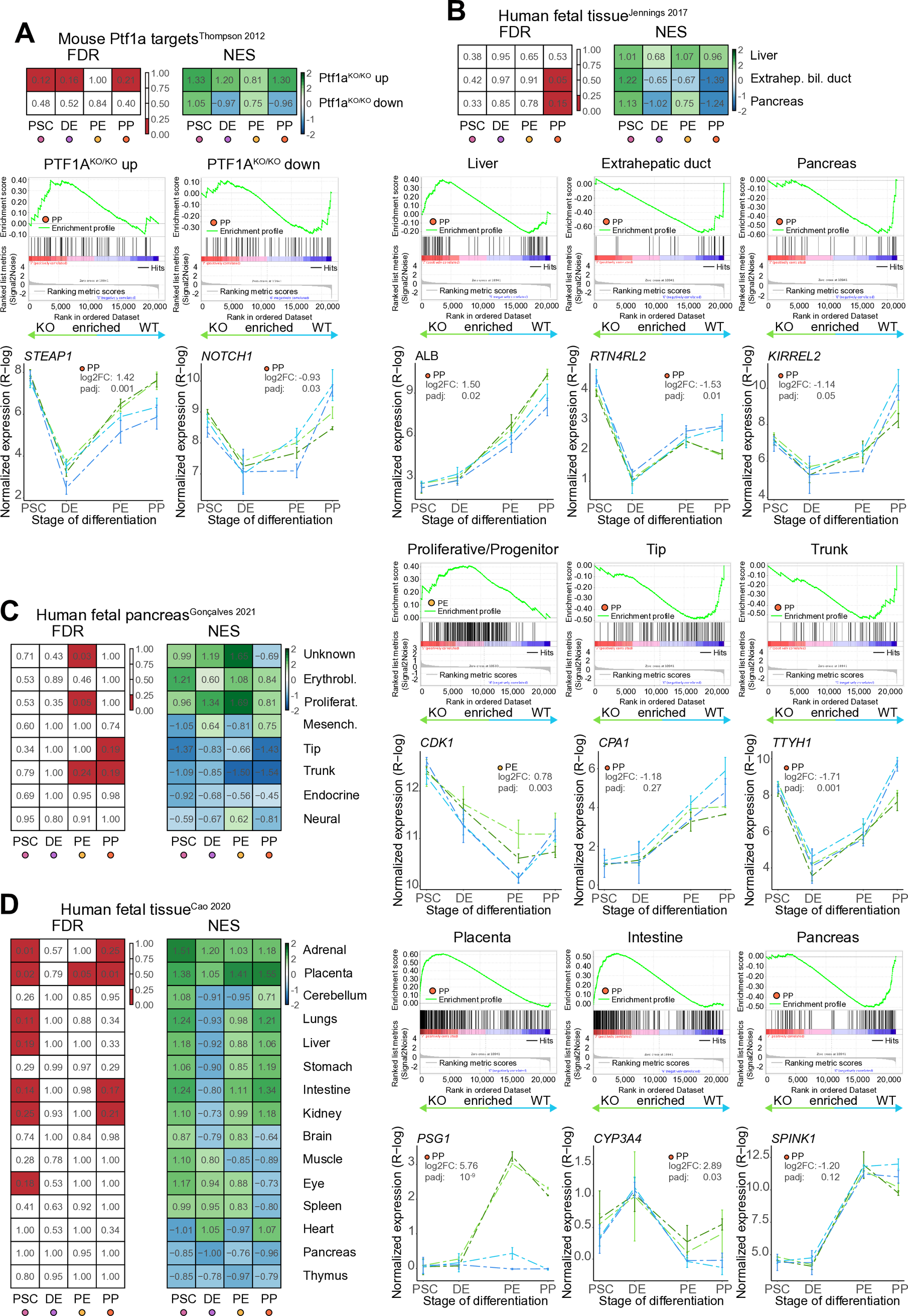

Supplement: S4 Fig — Gene set enrichment analysis (GSEA) for gene sets compiled from (A) an RNA microarray of Ptf1aKO/KO versus Ptf1aKO/WT murine pancreatic progenitors [31], (B) bulk RNA-seq from human fetal pancreas [29], (C) scRNA-seq from human fetal pancreas [73] and (D) scRNA-seq from human fetal tissues [30]. For all: False discovery rates (FDRs) ≤ 0.25 are highlighted in red (left panel), while comparisons that were enriched in PPDPFKO/KO cells are depicted in green and comparisons that were depleted in blue (right panel). GSEA plots against depicted reference gene sets are shown for the comparison of PPDPFWT/WT and PPDPFKO/KO PPs (only for proliferative progenitor of Gonçalves PE plotted). Line plots (Mean ± SEM) are displayed for genes, top ranked in GSEA. Additional information: For “PTF1AKO/KO upThompson 2012”, 4 out of 64 genes were significantly upregulated (DEGs) in PPDPFKO/KO PPs over PPDPFWT/WT PPs, while 0 were downregulated. For “PTF1AKO/KO downThompson 2012”, 0 out of 66 genes were up, 1 was down. For “liverJennings 2017”, 4 out of 79 genes were up, 2 were down. For “Extrahepatic biliary ductJennings 2017”, 1 out of 32 genes was up, 5 down. For “PancreasJennings 2017”, 0 out of 32 genes were up, 3 down. For “Proliferative/ProgenitorGonçalves 2021”, 2 out of 195 genes were up, 1 was down (at PE stage). For “TipGonçalves 2021”, 0 out of 111 genes were up, 1 was down. For “TrunkGonçalves 2021”, 0 out of 55 genes were up, 2 were down. For “Placenta PancreasCao 2020”, 20 out of 307 genes were up, 4 were down. For “Intestine PancreasCao 2020”, 7 out of 239 genes were up, 0 down. For “PancreasCao 2020”, 0 out of 107 gene were up, 1 was down. (TIF) [file pgen.1011657.s004.tif]

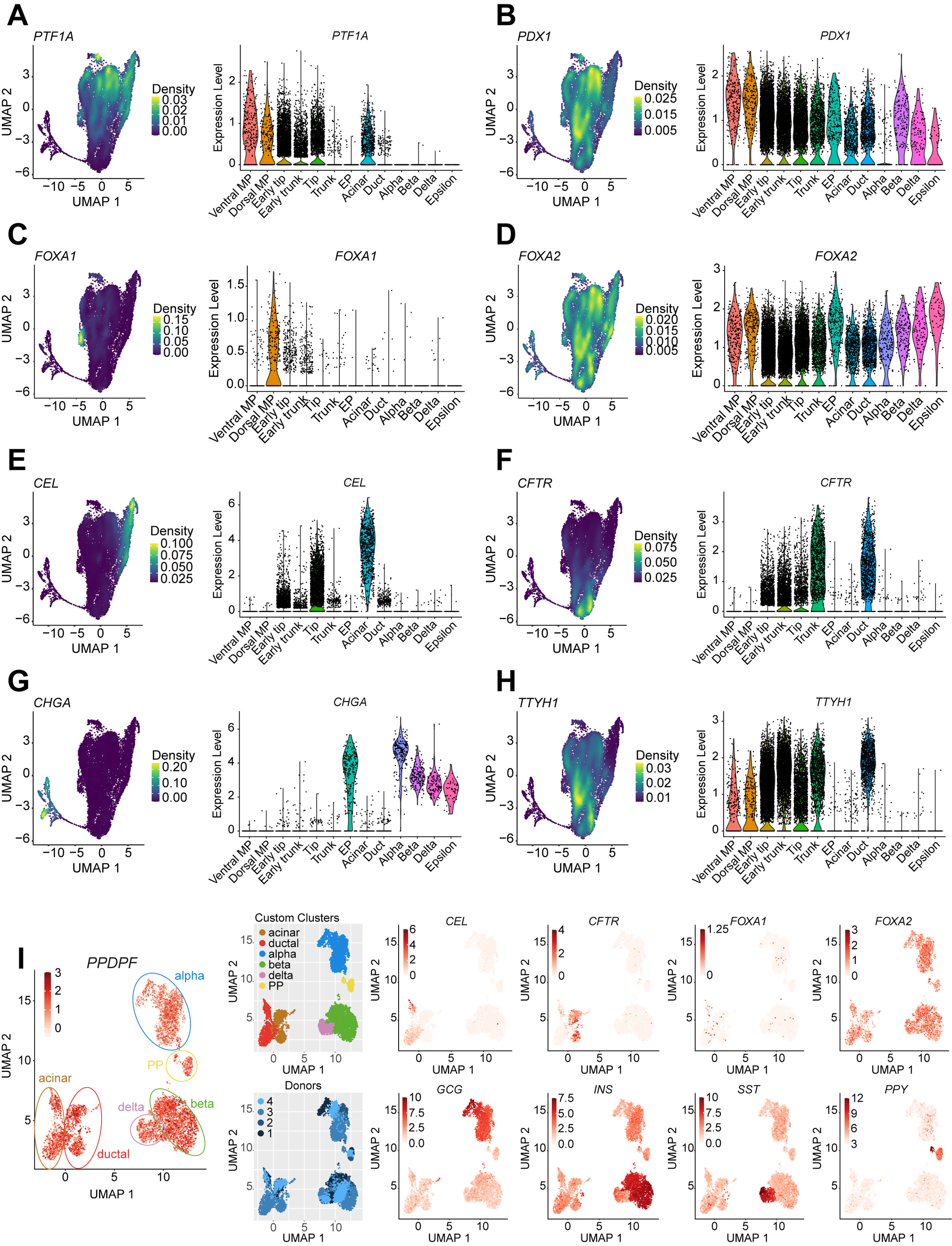

Supplement: S5 Fig — UMAP representations and violin plots for expression of (A) progenitor marker PTF1A, (B) PDX1, (C) FOXA1, (D) FOXA2, (E) acinar marker CEL, (F) ductal marker CFTR, (G) endocrine marker CHGA, and (H) trunk (and tip) marker TTYH1 in human fetal pancreas [32] complementing main Fig 8A-D. (I) Expression of PPPDF and selected cell type marker in human adult pancreas [33] revealing relatively broad expression of PPDPF. EP: endocrine progenitor; MP: multipotent progenitor; UMAP: Uniform Manifold Approximation and Projection. (TIF) [file pgen.1011657.s005.tif]

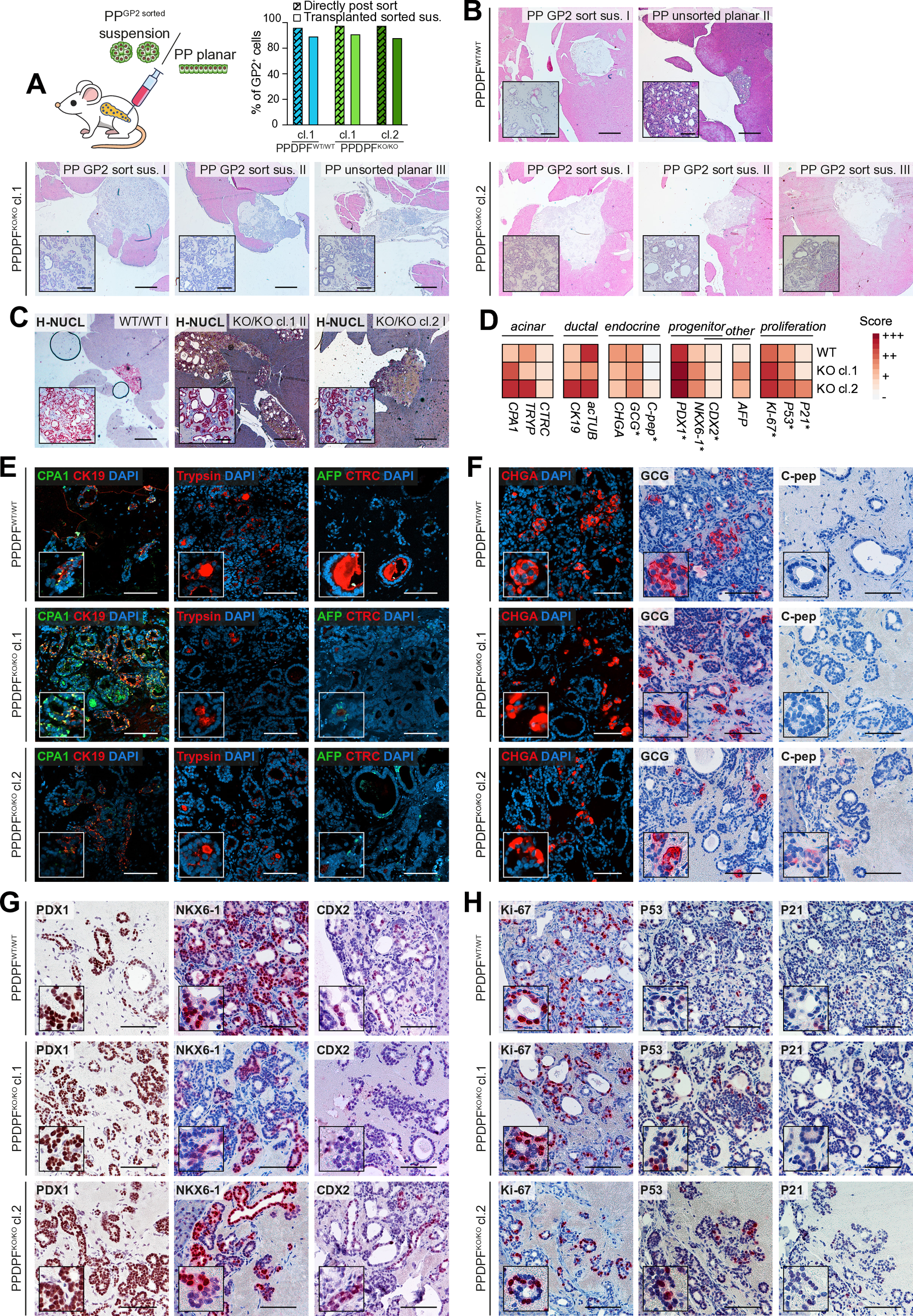

Supplement: S6 Fig — (A) Schematic overview of orthotopic transplantation experiments and flow cytometry-based quality control of GP2 sorting (n = 1). (B) Hematoxylin and eosin (H&E) staining of identified grafts with n = 2 for PPDPFWT/WT PPs and n = 3 for PPDPFKO/KO cl.1 and cl.2 PPs. Unsorted planar PPs were used when cell numbers after GP2 sort were not sufficient. (C) Human nucleoli (H-NUCL) staining illustrates human engraftment. (D) Quantification of depicted marker expression averaged over individual grafts of each genotype (− completely or nearly completely absent; + weak expression; ++ moderate expression; +++ strong expression. (E) Immunofluorescence (IF) staining of acinar marker CPA1, Trypsin, and CTRC, ductal marker CK19, and liver marker AFP. (F) IF and immunohistochemistry (IHC) of ductal cilia marker acetylated tubulin (acTUB), endocrine marker CHGA, GCG, and C-peptide (C-pep). (G) IHC of progenitor marker PDX1, NKX6-1, and CDX2. (H) IHC of proliferation marker Ki-67 and effectors of growth restriction P53 and P21. For all: Images are depicted that show strong marker expression to highlight trilineage competence. Scale bar: B-C: 500 µm, inlet: 100 µm. E-H: 100µm, inlets 2x enlarged. (TIF) [file pgen.1011657.s006.tif]
