# Supplementary material for: PPDPF is not a key regulator of human pancreas development
Source: PLoS Genet. 2025 Apr 7;21(4):e1011657. doi: 10.1371/journal.pgen.1011657 (PMC12037078; doi:10.1371/journal.pgen.1011657)
Supplement: Attachment — Submitted filename: Point-by-point-response_2024-12-20.pdf [file pgen.1011657.s014.pdf]

Please allow us in the following to address the reviewers' concerns point-by-point:

**Reviewer #1:**

**Minor Points:**

***Comment 1: Page 9, paragraph 2, line 8. Were these the hPSCs or the hESC cell line clones? The presumption is that you are referring to the hESC clones and not the hPSCs described in the prior paragraphs (as indicated in figure 3).***

**Answer to Comment 1**: We thank the reviewer for pointing out a potential misunderstanding in the text. Please note that we only used hESC lines and no induced pluripotent stem cells (iPSCs) throughout the study. hESCs are a subtype of hPSCs but we acknowledge the potential for misunderstanding. For consistency, we have revised the text to always write "hESCs" when specifically referring to the PPDPF KO and WT HUES8 cell lines. We maintained the use of stage labelling (hPSC, DE, PE; PP stage) throughout the manuscript.

Revised: While PPDPF-deficient hESCs differentiated into KIT$^+$/CXCR4$^+$ DE and PDX1$^+$ PE with similar efficiencies as their wildtype (WT) controls […]

---

***Comment 2: Page 16, paragraph 1, second-to-last line: "associated SNPs did not correlate with actual diabetes risk but not with actual diabetes risk" is confusing and perhaps a mis-typed sentence.***

**Answer to Comment 2**: We thank the reviewer for identifying this mis-typed sentence. We have revised the sentence which now reflects our intended meaning that the PPFPF-associated SNPs correlate with glycaemic alterations rather than diabetes risk.

**Revised:** Importantly, the PPDPF-associated SNPs did not correlate with actual diabetes risk but only with glycaemic alterations, thereby supporting the less important role for pancreas development in humans than in zebrafish.

---

***Additional Consideration of Reviewer 1: One consideration could be to generate a PPDPF KO mouse model and evaluate pancreatic development in vivo***.

**Answer to Reviewer's Suggestion**: We appreciate the reviewer's suggestion to generate a PPDPF KO mouse to assess pancreatic development in vivo. While this is an interesting biological approach, the scope of the current study does not extend to this. Our extensive human data does not indicate a major role of PPDPF in pancreas development in higher vertebrates. Therefore, we likely will not invest the necessary resources (money, time, personnel) in generating a Ppdpf KO mouse model in future. We hoe the reviewer understands this limitation within the context of our study.

---

## Reviewer #2:

### Major Points:

***Comment 1:* The Western blot shown in figure 2D is of poor quality. It is difficult to tell is a PPDPF expression is present in the PP cells, or if is just background.**

**Answer to Comment 1**: We appreciate the reviewer's comment on the quality of the Western blot shown in Figure 2D. We agree that the initial image was of poor quality. To improve this, we conducted a new Western blot with a different antibody (Proteintech, Cat No. 19912-1-AP). Unfortunately, this antibody did not provide clear results as evidenced by the many unspecific bands shown in **Reviewer Figure 1** (upper panel). The antibody previously used (Atlas Antibodies, HPA040929) was unfortunately discontinued and therefore unavailable for further experiments. We want to stress that the in the manuscript included mass spectrometry data successfully demonstrates and increase in PPDPF expression from hPSC stage to pancreatic lineage entry (Figure 2C). Please also see the PPDPF Western blot of higher quality in Supplem. Figure 2F demonstrating successful ablation of PPDPF protein in generated cell lines. Here, the validated KO band (Suppl. Fig 2F) was detected at approximatively 17kDA, as predicted in silico. Given these challenges with antibody suitability, we have removed the initial Figure 2D from the manuscript to avoid further confusion. We have updated the manuscript and references accordingly to reflect these changes.

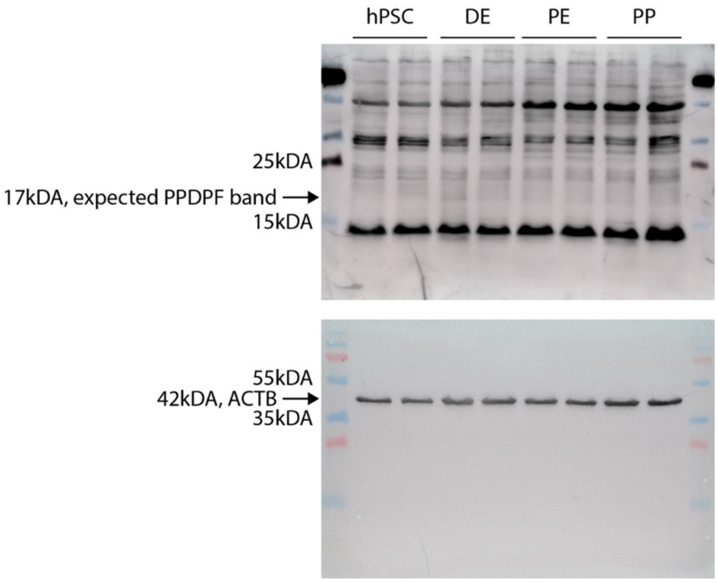


**Reviewer figure 1: PPDPF WB detection at PP stage.** 100µg of protein were loaded from HUES8 at pluripotent stem cell stage (hPSC), definitive endoderm (DE), pancreatic endoderm (PE) and pancreatic progenitor (PP) stage. ACTB (anti-b-Actin) has been applied as described in the initial manuscript Method section (Sigma, A5316, 1:5000). PPDPF (Proteintech, 19912-1-AP) was employed in a 1:500 dilution.

***Comment 2:* Is the promoter of PPDPF active at earlier stages than PP stage? The histone marks are shown for PP stage (Fig. 2F) but the data in Fig. 2C shows protein expression earlier at the PE stage?**

**Answer to Comment 2**: Thank you for raising this point. We would like to clarify that the promoter of PPDPF becomes increasingly active from earlier stages to the PP stage. In Suppl. Figure 1B, we show histone marks for earlier stages, including DE, GTE, PE, and PP. Specifically, the active histone mark H3K27ac shows a progressive increase from hPSC to DE, GTE, PE to PP stage. This supports the observed protein expression of PPDPF at the PE stage, as shown in Figure 2C, and aligns with the increasing activity of the PPDPF promoter.

***Comment 3: In figure 3 it is difficult to see that PDX1 staining in the immunofluorescence images, so these appear discordant with the flow cytometry results. Either better quality immunofluorescence images could be used to show PDX1 expression, or consider the use of single channel images.***

**Answer to Comment 3**: We agree with the reviewer's observation regarding the quality of the IF stainings in Fig.3. To address this, we performed new stainings from freshly started differentiations and replaced the previous IF images with higher quality ones in Fig. 3C. These updated images should now provide a clearer representation of PDX1 expression and align more closely with the flow cytometry results.

---

***Comment 4: The authors mention the upregulation of genes associated with placenta in the RNAseq data associated with Figure 6/Supp Figure 4. Considering that placenta is derived from the trophectoderm, and in Figure 2, the authors show 99.9% of cells are definitive endoderm, can the authors comment on this seeming discordance?***

**Answer to Comment 4**: We appreciate the reviewer's valuable feedback regarding the apparent discordance between the upregulation of placental genes in the RNA-seq data and the high proportion of DE cells observed in our differentiation protocol. While we achieved efficient DE differentiation, we acknowledge that in vitro differentiation systems may still exhibit some degree of impurity or cellular plasticity. This plasticity can lead to rare lineage bifurcations, where a small subset of cells may express markers almost completely absent under baseline conditions. It is important to note that, as shown in Suppl. Fig. 4D and Suppl. Table 3, the expression levels of the placental genes (scale of normalized expression in PSG1 plot) were generally very low across all stages. While the RNA-seq data showed a relative upregulation of some lineage markers (on transcriptome level), this should not be interpreted as a true lineage specification or commitment. This was further underpinned by our transplantation experiments, where neither PPDPF KO nor WT cells formed placenta-like structures, suggesting that the observed regulation at the RNA level does not translate into proper lineage commitment.To address the raised issue more directly, we now performed a follow-up PP differentiation experiment and analyzed PSG1/8 (high sequence overlap of these two genes) expression by qPCR and flow cytometry. We again observed a relative upregulation of *PSG1/8* upon PPDPF KO/KO at the mRNA level but, as shown in **Reviewer figure 2A**, the overall transcript levels were very low, and no protein expression was detected (**Reviewer figure 2B**). Therefore, we conclude that the upregulation of PSG genes observed in RNA-seq was statistically significant but not physiologically relevant, given the extremely low expression levels. We have adjusted the manuscript to clarify the observed upregulation in RNA-seq is not indicative of true placental specification, and to emphasize the very low expression levels of these genes, ensuring that this context is better communicated. We also note that the potential role of PPDPF in placenta development, if any, is outside the scope of this study and cannot be concluded from our findings.

**Reviewer table 1: Newly used qPCR primers**

| | |
|---|---|
| PSG1 qPCR fwd | CCCTGAATGTCCTCTATGGTCC |
| PSG1 qPCR rev | GTCCGCAGAACAGGACAAGT |

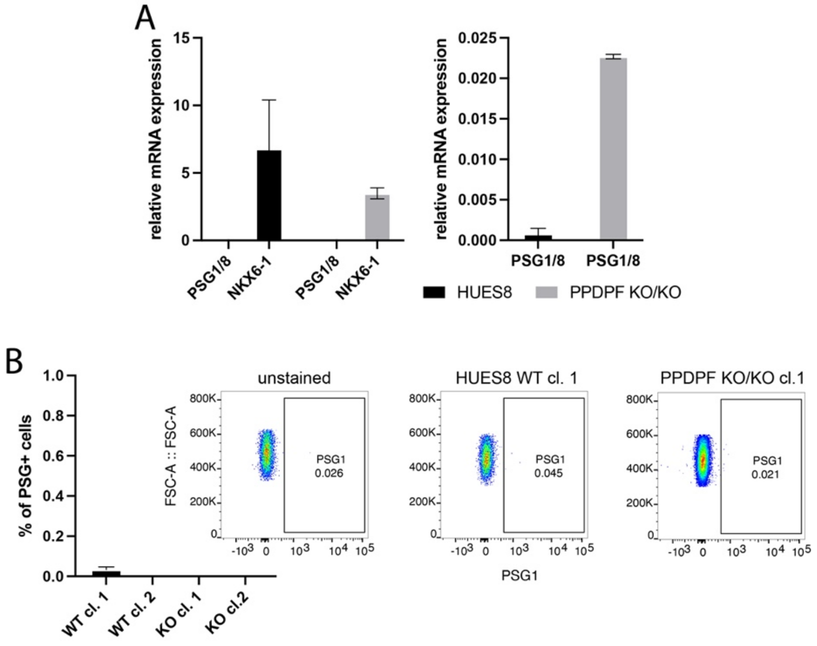


**Reviewer figure 2: Analyzing PSG1 expression at PP stage of pancreatic in vitro differentiation. A) qPCR validation for placental, *PSG1/PSG8*, gene expression.** RNA samples from 1 differentiation showing the Mean±SD of two clones. Each clone was measured in duplicates. qPCR results were normalized against the housekeeping gene HMBS. **B) Live cell flow cytometry against PSG1.** Samples from the same differentiation as (A) were harvested and stained according to manufacturer's recommendation with PSG1 antibody (Thermo, Catalog # MA5-38611, 1:200). Mean±SD of two independent wells of the same differentiation is plotted, relative to the negative control. We conclude that no PSG1-expressing cell subpopulation could be detected in any condition. Please note, that we could not implement a placental cell (positive) control in our test.

**Initial text related to comment 4:** In addition, human fetal pancreatic signatures including the more mature tip and trunk states were depleted (**Supplementary Figure 4B,C**), while several non-pancreatic tissue signatures such as intestine and most strongly, placenta were enriched (**Supplementary Figure 4D**). Related to latter, several pregnancy-specific glycoproteins (PSGs) were highly upregulated in PPDPF$^{KO/KO}$ PE/PP samples (**Supplementary Figure 4D** and **Supplementary Table 3**).

[…] The observed delay and reduction in the generation of hPSC-derived PPs could explain changes in glycaemic control, especially in co-occurrence with additional unknown (non-genetic) risk factors.

**Revised text according to comment 4:** In addition, human fetal pancreatic signatures including the more mature tip and trunk states were depleted (**Supplementary Figure 4B,C**), while several non-pancreatic tissue signatures such as intestine and ~~most strongly,~~ placenta were enriched (**Supplementary Figure 4D**). Related to latter, several pregnancy-specific glycoproteins (PSGs) were ~~highly~~ significantly upregulated in PPDPF$^{KO/KO}$ PE/PP samples, but their actual transcript levels remained extremely low (**Supplementary Figure 4D** and **Supplementary Table 3**).

[…] While initial *in vitro* differentiation experiments suggested a reduced capacity of PPDPF$^{KO/KO}$ hESCs to develop into PPs, global RNA-seq experiments revealed that stage-specific changes were rather small and might represent a slight delay in PP development together with a ~~selective~~ slight upregulation of non-pancreatic genes ~~such as placental PSG genes~~.

---

***Comment 5:* *The transcriptomic changes for NKX6-1 expression are very small (Fig. 6F) but the protein changes (Fig. 2B) are quite large. Can the authors address why they think this is seen?***

**Answer to Comment 5**: We appreciate the reviewer's observation regarding transcriptomic changes depicted in Fig.6. versus protein-level changes presented in Fig. 2B.

While the log2FC of -1.16 in bulk RNA-seq indicates a modest 2.23-fold reduction in NKX6-1 mRNA in PPDPF KO/KO cells, this finding is consistent with the qPCR results (Fig. 3D) , where we observed a more pronounced 4.58-fold reduction in mRNA levels from independently started differentiations. Additionally, flow cytometry analysis revealed a 2.53-fold reduction in the number of NKX6-1 positive cells. In addition, a discrepancy between changes between mRNA level and protein-might be attributed to several factors. Post-transcriptional regulation of gene expression, including mechanisms such as translation efficiency, protein stability, and cellular localization, could account for a greater change in protein levels. Given the different modalities of the measurement (mRNA/protein; sequencing/primer/antibody and importantly expression levels/number of positive cells), we consider our measurements as concordant supporting the in the manuscript postulated claims. We are therefore confident that these findings support our manuscript's conclusions and highlight complex regulatory mechanisms governing NKX6-1 expression.

---

***Comment 6:* *In figure 7a, cell counts are performed to assess total cell number. At the PE stage, the PPDPF ko cell lines shown a significant increase in cell number; however, the are no significant changes to either cell death or proliferation (Fig 7b-g). Can the authors comment on this?***

**Answer Comment 6**: We appreciate the reviewer's careful observation. This finding was also unexpected for us. While we cannot definitely resolve whether the measurements at d0, d4, d10, and d14 were too static to capture more subtle temporal changes in proliferation or apoptosis during DE to PP differentiation, we wish to emphasize that the observed temporary increase in PPDPF KO/KO PE cell numbers does not suggest a pro-proliferative role of PPDPF during pancreatic in vitro differentiation. In light of this, we slightly have adapted the discussion in the manuscript to address the raised concern and clarify that these findings do not contradict the claims regarding the role of PPDPF in pancreatic differentiation.

**Revised text according to comment 6**: However, proliferation marker and growth characteristics appeared not decreased upon loss of PPDPF during all investigated stages of pancreatic development including in vitro differentiation into DE, PE, and PP stage and in vivo specification into acinar, ductal, and endocrine cells.

---

*Comment 7:* ***In the human fetal scRNAseq (Fig. 8C/D), very little change is seen in the expression level of PPDPF over time. Do the authors think the small differences between MP, endocrine and acinar cells (for example) is meaningful?***

**Answer to Comment 7**: We appreciate the reviewer's thoughtful comment on the small differences in PPDPF expression observed in human fetal scRNA-seq data. Indeed, we were also surprised by the low changes in *PPDPF* expression across different developmental stages and cell types. This finding further supports our conclusions in the study, where we did not observe a highly acinar-specific role for PPDPF, in contrast to previous results in zebrafish. Since we did not detect substantial developmental defects upon PPDPF ablation, we do not consider the observed small changes in expression levels to be biologically meaningful for the investigated aspects.

**Revised text according to comment 7:** […] its broad scRNA-seq expression profile in the pancreas however appears similar to FOXA2 expression (**Supplementary Figure 5**) rendering a sole dependence on PTF1A and acinar-specific function unlikely.

---

*Comment 8:* ***Does GP2 expression vary between wt and PPDPF ko cells during PP stage or at time of transplant?***

**Answer Comment 8**: We thank the reviewer for that well-considered question regarding GP2 levels for transplanted cells and at PP stage. In Figure 3, we did not observe differences of *GP2* mRNA expression between PPDPF$^{WT/WT}$ and PPDPF$^{KO/KO}$ PPs. This might be partly due to overall rather low levels of GP2 that are achieved with the applied protocol. The very low number of GP2 positive cells in **Reviewer Figure 3**, where we now performed a new differentiation experiment, do not allow to draw meaningful conclusions. Please note that transplantation experiments were performed using the newest PP protocol providing higher yields of differentiation efficiency (Merz, et al. [1]). At the time point of transplantation of PE cells (Figure 9), no GP2 expression can be expected, since GP2 marker expression is appearing between PE and PP stage. PP cells were sorted prior to transplantation and comparable GP2 levels of transplanted PP cells are shown in **Supplement Figure 6A**.

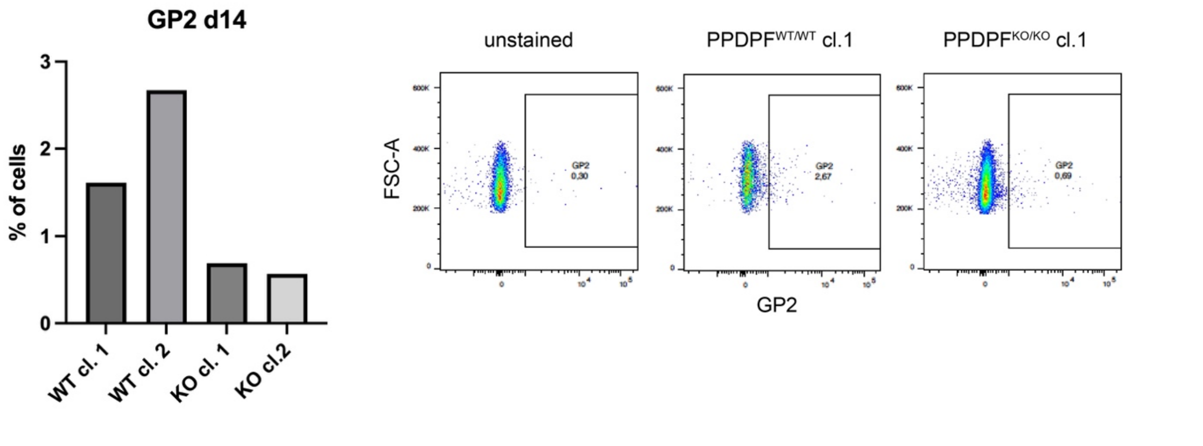


**Reviewer figure 3: GP2 protein measurements by FC.** Results from one differentiation are plotted in a bar graph (left) with applied gating strategy (right).

---

***Comment 9:* No control/isotype staining for flow cytometry analysis is shown, and as such, the validity of the gating is unable to be assessed. Additionally, there is no mention of how live/dead cell selection occurred. Could these details as well as the control stains please be shown.**

**Answer Comment 9**: We thank the reviewer for this important comment and have included control stains of FC data directly in the figures (Figure 3A, Figure 7B-F, Suppl. Figure 2G, Suppl. Figure 6 A). Please note that we used 2nd AB controls for not directly fluorophore-conjugated antibodies and unstained controls for directly fluorophore-conjugated antibodies. All flow cytometry stainings that are depicted in the manuscript are well established stainings that work consistently and have been established with additional controls including isotype control during the initial establishment in the lab.
Regarding the second aspect of comment 9, how live/dead cell selection was performed: For surface marker flow cytometry, 150ng/µL DAPI was added after the last washing step. For live/dead cell selection for AnnexinV flow cytometry, 7AAD was used following recommendations of the kit manufacturer (Biolegend, 640922). We have clarified the Method section accordingly.

**Revised text to comment 9:** DAPI was added after the last washing step at a concentration of 150 ng/µl to distinguish viable (DAPI$^-$) and dead (DAPI$^+$) cells during analysis.

We have additionally added an analysis section (also see missing information under minor points):

**Revised text to second part of comment 9 (i.e. Flow cytometry analysis)** Analysis of flow cytometry data including gating was performed using FLowJo™ version 10.8.1. Briefly, Cells were gated on the area of forward and sideward scatters. Single cells were subsequently defined by the area and width of the forward scatter followed by an additional stringency step using the area and width of the sideward scatter. In case of live cell measurements, DAPI$^-$ or 7AAD$^-$ cells were gated as living cells for subsequent marker analysis (area of specific channels).

---

***Comment 10:* Overall, the authors conclude that PPDPF knockout results in a delay in differentiation rather than a difference in differentiation capacity. If the PP stage cells were further differentiated in vitro to an endocrine cell stage, does the delay continue? At what point (if any) do the knockout cells catchup to the wild-type cells?**

**Answer to comment 10**: We thank the reviewer for this insightful comment regarding the temporal aspects of PPDPF KO cells during pancreatic differentiation. As the reviewer pointed out, we did not fully dissect whether delay in differentiation observed at PP stage continues during further in vitro differentiation to an endocrine cell stage or whether the KO cells eventually catch up to their WT counterparts. Initially, we described a developmental delay based on the persistent expression of genes and gene sets characteristic of earlier stages at PPs. In RNA-seq analysis of Figure 5C for example, we observed that some of the PPDPF KO/KO PP samples clustered more closely with WT PE cells than WT PP cells. To better characterize this delay, we performed gene set enrichment analysis for developmental signatures of all key differentiation stages up to the PP stage (Figure 6A). This analysis revealed that relative to the WT counterparts, PPDPF KO/KO PE and PP cells were not only depleted of pancreas-specific gene sets (such as those for pancreatic endoderm, PE and

pancreatic progenitor, PP) but were also enriched for the earlier stages gene sets, most notably definitive endoderm, DE. This finding further supported an incomplete differentiation. We acknowledge that while these results indicate incomplete differentiation into PPs, they do not provide direct evidence of a delay or "catch up" effect at later stages. Consequently, we have revised the wording in the manuscript to reflect this clarification and avoid misleading interpretation of a "catch-up" phase prior to PP stage.

**Initial text to comment 10:**

**- RNA-seq analysis reveals developmental delay and tissue bifurcation upon PPDPF ablation.**

- In hierarchical clustering, 4 of 8 KO samples clustered even closer to PE samples than to the other PP samples suggesting some degree of developmental delay in PPDPFKO/KO PPs (Figure 5C).
- Until now, we observed a reduction in PPs in *in vitro* differentiation experiments of PPDPFKO/KO PSCs and RNA-seq experiments revealed some degree of developmental delay and lineage bifurcation into non-pancreatic tissue.
- While initial in vitro differentiation experiments suggested a reduced capacity of PPDPFKO/KO hESCs to develop into PPs, global RNA-seq experiments revealed that stage-specific changes were rather small and might represent a slight delay in PP development together with a selective upregulation of non-pancreatic genes such as placental PSG genes.

- Figure 6: Delay of pancreatic progenitor differentiation upon PPDPF ablation.

- PE- and PP-specific transplantation experiments finally disclosed that PPDPF$^{KO/KO}$ hPSCs indeed possess the competence to develop into all major cell types of the human fetal pancreas.

**Revised text to comment 10:**

**- RNA-seq analysis suggests incomplete PP differentiation and tissue bifurcation upon PPDPF ablation.**

- In hierarchical clustering, 4 of 8 KO samples clustered ~~even~~ closer to PE samples than to the other PP samples suggesting an incomplete differentiation into PPs upon PPDPF ablation (Figure 5C).
- Until now, we observed a reduction in PPs in *in vitro* differentiation experiments of PPDPF$^{KO/KO}$ hESCs and RNA-seq experiments revealed the preservation of endodermal gene expression and a lineage bifurcation into non-pancreatic tissue, however, both at relatively low expression level.
- The observed ~~delay and~~ reduction in the generation of hPSC-derived PPs could explain changes in glycaemic control, especially in co-occurrence with additional unknown (non-genetic) risk factors.

- Figure 6: Incomplete pancreatic progenitor differentiation upon PPDPF ablation.

- PE- and PP-specific transplantation experiments and *in vitro* islet differentiations finally disclosed that PPDPF$^{KO/KO}$ hESCs indeed possess the competence to develop into all major cell types of the human fetal pancreas.

While we observed that PPDPF-ablated PEs and PPs formed pancreatic-like tissue comparable to PPDPF-proficient cells, we had not initially analyzed the temporal aspects of the lineage specification. To address the reviewer's question about when PPDPF KO/KO PPs catching up during their specification into more mature stages, we performed an additional endocrine in vitro differentiation experiments as suggested by Reviewer 2.
In a proof-of-concept 34-day experiment, we analyzed PDX1/NKX6-1/INS and GCG/C-peptide levels by flow cytometry at day 13 (PP stage), d20, d27 and d34 and analzed the endpoint by additional immunofluorescence staining. We observed comparable levels of all markers as early as day 20, substantiating that PPDPF KO/KO PP cells are not substantially impaired in their pancreatic specification capacity. The results of this cost-intensive experiment (high well formats for suspension cultures needed for the readouts), where we have differentiated two independent clones of each genotype until d20 and followed up with one clone per genotype until the final d34 endpoint have been included in a new Figure 10.

We have therefore added following paragraph in the results section:

PPDPF$^{KO/KO}$ cells efficiently differentiate into endocrine islet-like organoids in vitro.

In a nutshell, observed differences between PPDPF proficient and deficient cells to form PP cells did not perpetuate to more mature pancreatic stages in vivo. It remained, however, unclear when PPDPF$^{KO/KO}$ PPs might had caught up to PPDPF$^{WT/WT}$ during PP specification. To dissect temporal aspects more closely, we differentiated PPs in vitro into endocrine islet-like organoids(36, 37) in a proof-of-concept experiment over 34 days (Figure 10A). hPSCs were first differentiated into PP cells, where the decrease in NKX6-1+ PPs upon PPDPF ablation was again reflected (Figure 10B, left), and no substantial premature activation of endocrine marker was found (Figure 10B, right). In contrast, upon further differentiation into endocrine progenitors (Figure 10C), pre-islets (Figure 10D) and more mature islet-like organoids (Figure 10E), the number of derived alpha-like cells (GCG) and beta-like cells (C-peptide, INS) was highly similar. Comparable endocrine differentiation efficiencies between PPPDF$^{WT/WT}$ and PPDPF$^{KO/KO}$ cells were further validated by immunofluorescence imaging on paraffin-embedded and sectioned islet-like organoids (Figure 10F). We therefore concluded that PPDPF$^{KO/KO}$ PPs harbour the capacity to efficiently specify pancreatic endocrine cells *in vitro* with no differences on marker expression being observed from endocrine progenitor stage on day 20 on.

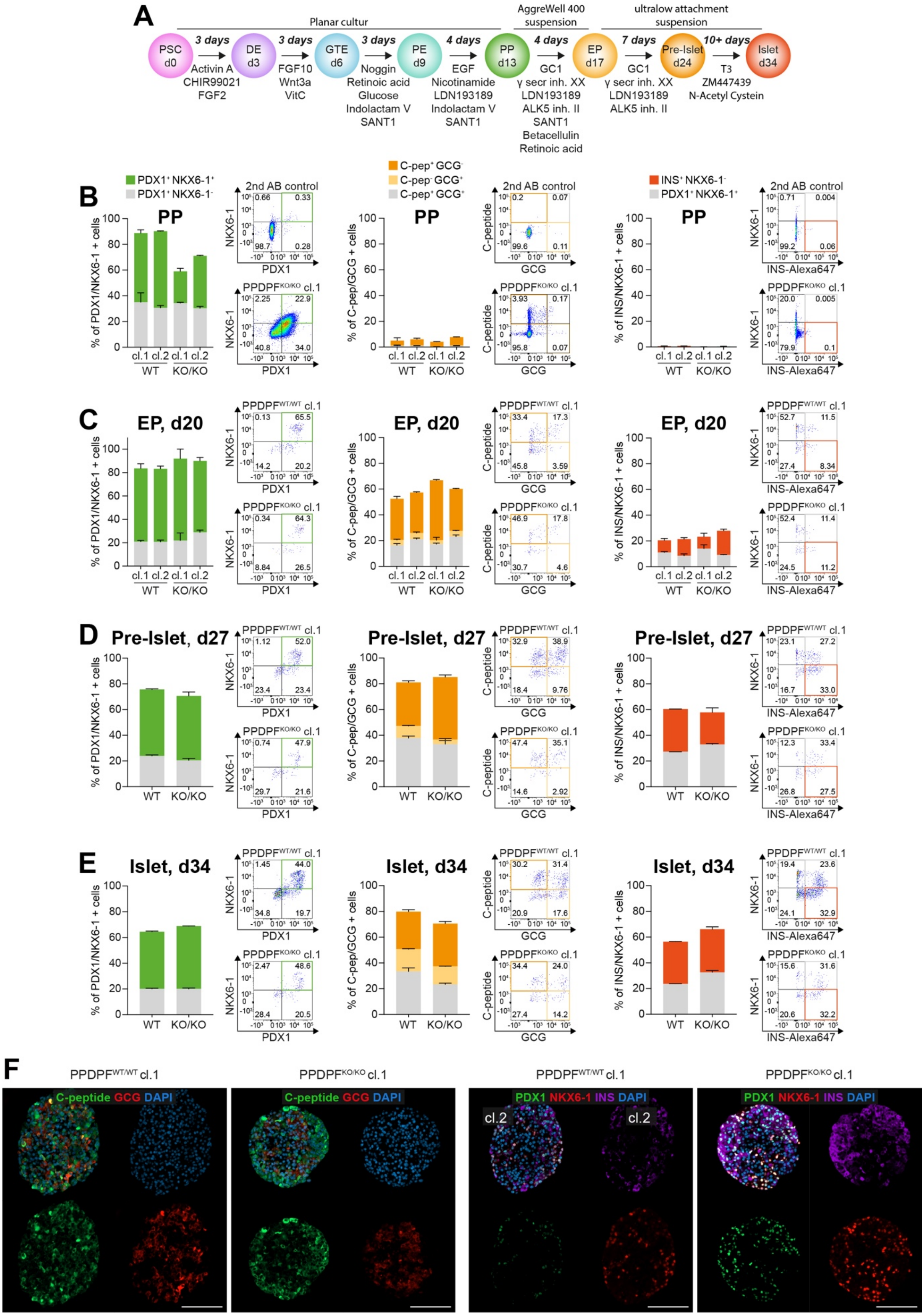


Figure 10: PPDPF$^{KO/KO}$ cells efficiently differentiate into endocrine islet-like organoids in vitro. (A) Schematic overview of human pluripotent stem (hPSC) cell-based differentiation into definitive endoderm (DE), gut tube endoderm (GTE), pancreatic endoderm (PE), and pancreatic progenitor (PP) cells in planar cultures followed by

3D endocrine progenitor (EP) formation and maturation into pre-islets and islet-like organoids at d34. (B-E) Differentiation efficiencies of clonal $PPDPF^{WT/WT}$ and $PPDPF^{KO/KO}$ hPSC lines, measured by flow cytometry (n=1 (independent differentiations), in duplicates (two wells per differentiation); Mean ± SD; at PP stage (B), EP stage (C), Pre-islet (D) and islet stage (E). (F) Representative immunofluorescence imaging of the same key endocrine marker (PDX1, NKX6-1, C-peptide, GCG, INS) on formalin-fixed paraffin-embedded (FFPE) organoid sections at d34 of differentiation.

Furthermore, we have updated parts of Methods section now including endocrine in vitro differentiation experiments.

**Initial text (part 2) to comment 10**

- Pancreatic differentiation for in vitro experiments
- Pancreatic differentiation for in vivo experiments

**Revised text (part 2) to comment 10**

- Pancreatic progenitor differentiation for in vitro experiments
- Pancreatic progenitor differentiation for in vivo experiments
- In addition, we included a new method section for the in vitro differentiation into endocrine organoids and updated the flow cytometry section and its employed antibodies:

In vitro differentiation into pancreatic endocrine organoids

We performed stem cell-derived islet differentiation as detailed in (63).

[…]

- *flow cytometry* […] Endocrine organoids were singularized with TrypLE for around 9-12 min with pipetting up and down in-between and control of proper singularization under the microscope. Planar cells were harvested and singularized as for surface marker staining.
- GCG (Sigma, G2654, 1:500), C-pep (Cell Signaling, 4593, 1:100), and INS-Alexa647 (Cell Signaling, 3014, 1:80).
- *IHC* […] Pancreata of transplanted mice and endocrine organoids were fixated with 4% paraformaldehyde solution (PFA) at 4°C o/n. […] For endocrine organoids, following ABs were used: PDX1 (R&D, AF2419, 1:500), NKX6-1 (DSHB, F55A12 (concentrate), 1:150), GCG (Sigma, G2654, 1:1000), C-pep (Cell Signaling, 4593, 1:200), and INS (Abcam, ab181547, 1:5000).

---

## Minor Points:

**Missing information:**

- GSE accession number for bulk RNA sequencing data generated currently missing

- Software used for image analysis/processing is missing from methods

- Software used for FACS plot generation is missing from methods

**Answer to Minor Points 10**: We thank the reviewer for this careful observation and have added the missing information accordingly.

- Own RNA-seq data (FASTQ file) and pre-processed files (read count and R log transformed count matrix) are deposited at Gene Expression Omnibus (GSE282625, reviewer access token: svilosqgpnyjhgv).

- We have also added an additional image analysis part:

**Revised according to "Minor" comment:**

[…] IF images were acquired on an Axio Observer 7 microscope (Zeiss) equipped with a Axiocam 702 camera (Zeiss) using ZEN blue software (Zeiss).

[…] IHC images were acquired on a DM5500B microscope equipped with a DMC5400 camera (Leica) using the Application Suite software (Leica).

[…]

ICC, IF, and IHC image analysis

For ICC/IF and IF, brightness and contrast was adjusted in ZEN blue (Zeiss) and for IHC in Photoshop (Adobe) according to good scientific practice. Images were then exported as jpeg files and cropped in Adobe Illustrator (Adobe) for final image compilation.

---
